# HOXC6 impacts epithelial-mesenchymal transition and the immune microenvironment through gene transcription in gliomas

**DOI:** 10.1186/s12935-022-02589-9

**Published:** 2022-04-29

**Authors:** Hui Huang, Zhengyuan Huo, Jiantong Jiao, Wei Ji, Jin Huang, Zheng Bian, Bin Xu, Junfei Shao, Jun Sun

**Affiliations:** Department of Neurosurgery, Wuxi People’s Hospital Affiliated to Nanjing Medical University, No. 299 Qing Yang Road, 214023 Wuxi, Jiangsu China

**Keywords:** HOXC6, Glioma, EMT, Immune, Tumour microenvironment, Biomarker

## Abstract

**Background:**

Gliomas are the most common primary malignant tumours of the central nervous system (CNS). To improve the prognosis of glioma, it is necessary to identify molecular markers that may be useful for glioma therapy. HOXC6, an important transcription factor, is involved in multiple cancers. However, the role of HOXC6 in gliomas is not clear.

**Methods:**

Bioinformatic and IHC analyses of collected samples (n = 299) were performed to detect HOXC6 expression and the correlation between HOXC6 expression and clinicopathological features of gliomas. We collected clinical information from 177 to 299 patient samples and estimated the prognostic value of HOXC6. Moreover, cell proliferation assays were performed. We performed Gene Ontology (GO) analysis and gene set enrichment analysis (GSEA) based on ChIP-seq and public datasets to explore the biological characteristics of HOXC6 in gliomas. RNA-seq was conducted to verify the relationship between HOXC6 expression levels and epithelial-mesenchymal transition (EMT) biomarkers. Furthermore, the tumour purity, stromal and immune scores were evaluated. The relationship between HOXC6 expression and infiltrating immune cell populations and immune checkpoint proteins was also researched.

**Results:**

HOXC6 was overexpressed and related to the clinicopathological features of gliomas. In addition, knockdown of HOXC6 inhibited the proliferation of glioma cells. Furthermore, increased HOXC6 expression was associated with clinical progression. The biological role of HOXC6 in gliomas was primarily associated with EMT and the immune microenvironment in gliomas. High HOXC6 expression was related to high infiltration by immune cells, a low tumour purity score, a high stromal score, a high immune score and the expression of a variety of immune checkpoint genes, including PD-L1, B7-H3 and CLTA-4.

**Conclusions:**

These results indicated that HOXC6 might be a key factor in promoting tumorigenesis and glioma progression by regulating the EMT signalling pathway and might represent a novel immune therapeutic target in gliomas.

**Supplementary information:**

The online version contains supplementary material available at 10.1186/s12935-022-02589-9.

## Introduction

Gliomas are the most common primary malignant tumours of the CNS, CNSaccounting for 81% of intracranial malignant tumours in adults and 2% of all malignant tumours in the body. The incidence of glioma is approximately 4.67–5.73/100,000 [1]. As the most lethal glioma, glioblastoma accounts for 70–75% of all diffuse glioma diagnoses, and glioblastoma patients have a median overall survival (OS) of 14–17 months [2]. To address this problem and improve the prognosis of gliomas, it is necessary to identify molecular markers that may be useful for glioma therapy.

The homeobox (HOX) gene family, an evolutionarily highly conserved polygenic family, was first found to be involved in developmental regulation in *Drosophila*. In humans, the genes have been reported to be an important family of genes that regulate embryonic development as well as cell growth and differentiation *in vivo* [3]; these genes have been implicated in numerous tumours. As members of the HOX family, it has been reported that the HOXA7 and HOXA9 genes are vital for establishing and maintaining aberrant HOXA9-HOXA13 gene expression in patients with acute myeloid leukaemia [4]. Many studies have shown that HOXD3 and HOXB13 are upregulated in breast cancer patients [5]. HOXB5, one of the HOXB clusters, is overexpressed in retinoblastoma cell lines and tissues [6].

In recent years, an increasing number of studies have confirmed that HOXC6 is involved in many physiological and pathophysiological processes, such as those in nasopharyngeal carcinoma [7], gastric cancer [8], oesophageal squamous cell carcinoma [9], and prostate cancer [10]. In addition, HOXC6, under the regulation of several signalling pathways, including the TGF-β pathway [11] and Wnt pathway [12], is highly expressed and associated with promoting in tumours [12]. Therefore, HOXC6 represents a potential factor affecting the tumour microenvironment. Immune checkpoint blockade has provided new insights into immunotherapy of gliomas [13]. Nevertheless, because of tumour heterogeneity and immune escape, a detectable clinical benefit from immunotherapy is not achieved in all patients [14, 15]. Thus, new biological targets are urgently needed.

In this study, we downloaded HOXC6 expression data from The Cancer Genome Atlas (TCGA), the Chinese Glioma Genome Atlas (CGGA) and the Rembrandt database and analysed the abnormal expression and prognostic value of HOXC6 in glioma patients based on data from these publicly available databases. Additionally, the correlation of the immune microenvironment was explored using the UCSC XENA and TISIDB databases. Our research focused on HOXC6 as a key factor influencing the EMT-related invasion and migration of gliomas and an immune-related biomarker.

## Methods and materials

### Patient data collection

The gene expression and clinicopathological information in TCGA and the Genotype–Tissue Expression (GTEx) database were obtained from the UCSC XENA public repository (https://xenabrowser.net/datapages/), CGGA database (http://www.cgga.org.cn/) and Rembrandt database supplied by gliovis (http://gliovis.bioinfo.cnio.es/).

### Clinical samples and immunohistochemistry (IHC)

Tissue microarray chips containing samples from a total of 299 patients were obtained from the affiliated hospitals and Shanghai Outdo Biotech Company. The follow-up data for 176 of these 299 patients were used for subsequent survival analysis. All patient information was obtained and used in accordance with the approved protocols of the institutional review boards of the participating institutions. Tissue slides were incubated with rabbit anti-HOXC6 antibody (1:500, Santa Cruz, sc-376330, America), anti-rabbit secondary antibody from Zymed Systems (InvitrogenCA) and 3,3’-diaminobenzidine to visualize IHC labelling. The slides were lightly counterstained with crystal violet. Normal rabbit IgG was used to verify the specificity of the IHC labelling. The results were analysed by ImageProPlus software (version 6.0). We determined the positive expression intensity of IHC by employing the mean optical density [16], which indicates the average reaction intensity of all selected objects in a field of vision. The mean optical density was calculated using the IOD Sum/Area Sum method. To ensure the authenticity of the measurement results, we adjusted the optical density of all the images and set identical parameters in HIS mode prior to measurement.

### Pan-cancer analysis of HOXC6 mRNA expression

HOXC6 expression data across different cancers compared with normal tissues were downloaded from Oncomine (www.oncomine.org) based on a |fold change|>1.5, p value < 0.001 and top 10% gene ratio. Table 1 provides information about the datasets that met these thresholds. Expression data of 33 types of cancer vs. normal tissue were obtained from the TCGA and GTEx databases. Univariate hazard ratios for each cancer were also analysed and presented with forest plots.

**Table 1:** The Significant Changes in HOXC6 Expression at the Transcription Level between Different Types of Brain and CNS Cancers vs. Normal Brain Tissues (Oncomine Database)

Type of brain and CNS cancer versus normal brain tissues	Fold Change	P Value	T Test	Reference
Glioblastoma	2.448	2.26E-13	8.604	Murat Brain Statistics
Anaplastic Oligoastrocytoma	1.73	7.70E-04	5.609	Bredel Brain 2 Statistics
Glioblastoma	1.533	1.43E-08	8.502	Bredel Brain 2 Statistics
Glioblastoma	1.939	5.54E-08	7.565	Lee Brain Statistics
Glioblastoma	1.972	1.24E-16	9.853	Sun Brain Statistics
Anaplastic Astrocytoma	1.691	8.17E-05	4.527	Sun Brain Statistics
Brain Glioblastoma	2.714	6.23E-09	14.195	TCGA Brain Statistics

### Correlation with clinicopathological features and prognosis analysis

The relationships betweenHOXC6 mRNA expression and different clinicopathological features were analysed based on TCGA, CGGA, and Rembrandt data and clinical samples. Kaplan–Meier analysis and multiple Cox regression analysis of the prognostic effect of HOXC6 mRNA expression were performed by using the survival and survminer R packages; correspondingly, the receiver operating characteristic (ROC) curve was analysed by using the timeROC R package. The nomogram and calibration plots were constructed using the RMS package of R software.

### Bioinformatics analysis

We screened HOXC6 positively correlated genes (Spearman’s coefficient > 0.3, p < 0.05) from glioma data in public databases and a list of HOXC6 target genes from ChIP-seq. Based on their intersection, GO analyses were employed by using the R package clusterProfiler [17]. GSEA was performed on the basis of HOXC6 expression in TCGA samples [18]. Metagenes of immune infiltrating cells were downloaded from TISIDB (http://cis.hku.hk/TISIDB/index.php), and single-sample gene set enrichment analysis (ssGSEA) was conducted via the GSVA R package. All association analyses were investigated using Spearman correlation analysis. Stromal, purity and immune scores were available in the UCSC XENA public repository. The packages ggplot2, ggradar, UpSetR, and ComplexHeatmap in R software were used for data visualization. The analysis employed a list of immune checkpoint proteins gathered from an important previous study [19].

### Cell culture and transfection

U251, T98G, U118 and U87 human glioma cells and human astrocytes (HA) were cultured in Dulbecco’s modified Eagle’s medium (DMEM, Gibco, C11995500BT, Canada) with 10% foetal bovine serum (FBS, Gibco, 10,091,148, Canada) and supplemented with 1x penicillin/streptomycin (Gibco, 15140-122, Canada). All cultures were maintained in a 37 °C, 5% CO2 incubator (TFS3111, America).

The overexpression lentiviral vector for HOXC6 was constructed by Genechem (GXDL0168387, China). The sequences were cloned into the lentiviral vector GV358 with a Flag-tag. A lentiviral HOXC6 short hairpin RNA (shRNA) was purchased from Genechem (GIEL0177253, China). The shRNA sequence targeting human HOXC6 complementary DNA was 5’-GACCAGAAAGCCAGTATCCAG-3’. A scrambled shRNA was included as a negative control (NC). The target sequence was inserted into the GV248 lentiviral vector (Genechem, China).

### Western blot analysis

In this study, RIPA buffer (Cell Signaling Technology, 9806, America) was used to extract proteins from cells or tissues. Approximately 25 µg of the protein per lane was detected according to a standard protocol of sodium dodecyl sulfate (SDS)-polyacrylamide gel electrophoresis. The membranes were incubated overnight at 4 °C with HOXC6 (1:500, Abcam, ab151575, Britain) antibody. GAPDH (1:10000, Proteintech, 60004-1-Ig, China) was used as a protein loading control.

### RT q-PCR

Total RNA was extracted from cells using TRIzol Reagent (Sigma–Aldrich, T9424, America) in a ventilator according to the manufacturer’s guidelines. RNA was reverse-transcribed into cDNA using the PrimeScript RT Reagent Kit (Takara, RR047, Japan). Quantitative real-time PCR was performed using SYBR Premix Ex Taq™ II (Takara, RR047, Japan) in an ABI StepOnePlus system. The primers were provided by Sheng Kong Company, Shanghai, China. The sequences of the primers were as follows: HOXC6, CGCACAACTCTCTTTCACC and TCACTTGGAGGGCAATCT; MMP9, CAGTACCGAGAGAAAGCCTATT and CAGGATGTCATAGGTCACGTAG; CDH1, AGTCACTGACACCAACGATAAT and ATCGTTGTTCACTGGATTTGTG; CDH2, CGATAAGGATCAACCCCATACA and TTCAAAGTCGATTGGTTTGACC; DKK1, TACCAGACCATTGACAACTACC and TCCATTTTTGCAGTAATTCCCG; PD-L1, GCTGCACTAATTGTCTATTGGG and CACAGTAATTCGCTTGTAGTCG; B7-H3, CTGACAGATACCAAACAGCTGG and CGAAATCCCGGATGCTCA. IDO, CTGCCTGATCTCATAGAGTCTG and TTGTGGTCTGTGAGATGATCAA. The expression of the target genes was normalized to GAPDH, which served as an internal control. The comparative CT (ΔΔCT) method was used to analyse fold changes.

### Cell proliferation assay

#### Cell counting Kit-8 assay

To measure cell proliferation, we used Cell Counting Kit-8 (CCK-8) (Dojindo, CK18, Japan). Both control and transfected cells were seeded into 96-well plates (Corning, 3599, America) at an initial density of 1000 cells/well. Then, CCK8 reagent (10 µl/well) was added to each well. Twenty-four hours after seeding, cell viability was analysed with a microplate reader (Thermo, Multiskan-Spectrum, America) using CCK8 reagent as described above. This was considered day 0. Later, cell viability was measured every 24 h. Cell growth was indicated by the fold change from day 0 to day 4 and was graphed.

#### Colony-forming assay

Control and treated cells were plated in a 6-well plate (Corning, 3516, America) at a density of 1000 per well. Then, the cells were continuously incubated for 2 weeks until an evident colony was observed. The colonies were fixed and stained using 4% paraformaldehyde (Biosharp, BL538A, China) for 15 min and 0.1% crystal violet (BBI, E607309, China) for 30 min at room temperature. After photographs were taken, the total number of colonies was counted by using ImageJ software.

#### EdU assay

The EdU assay was used to detect the proliferation of control and treated cells. The cells were cultured for 1 h at 37 °C with EdU (Invitrogen, C10639, Canada) at a final concentration of 10 µM. The medium was removed, and the rest of the cells were fixed in 4% paraformaldehyde at room temperature for 30 min and then permeabilized with 1% Triton X-100 (Biosharp, BS084, China) for 20 min. The cells were then stained according to the manufacturer’s protocols and imaged by a fluorescence microscope (Leica Microsystems GmbH, Mannheim, Germany). Image synthesis and cell counting were performed using Fiji ImageJ. The number of EdU + cells divided by the number of DAPI + cells was the representation of the growth rate as well as proliferation.

### Chromatin immunoprecipitation (ChIP) and sequencing

We transfected U251 cells with the HOXC6 overexpression lentiviral vector with a flag tag. Then, ChIP was performed using the SimpleChIP Enzymatic Chromatin IP Kit (Cell Signaling Technology, 9003, America) according to the manufacturer’s instructions. The antibody DYKDDDDK (Cell Signaling Technology, 14,793 S, America) was used to pull down HOXC6 in the positive control and NC. Purified ChIP-DNA was verified on an agarose gel to ensure proper fragmentation and then sent to the company for ChIP-seq. After that, we used FastQC and MultiQC software for follow-up data quality control. Bowtie2 software was used for genome sequence alignment, and Picard was used to remove duplicate reads. MACS2 software was used for peak calling analysis, and the R package cheappeakAnno and MEME software were used for DNA binding motif enrichment analysis for annotation and statistical assessment of the analysis results [20]. Then, we performed integrative analysis on HOXC6 differentially regulated genes and ChIPseq annotated targets [21] (Additional file 1).

## Results

### Overexpression of HOXC6 in Gliomas

**Fig. 1:**
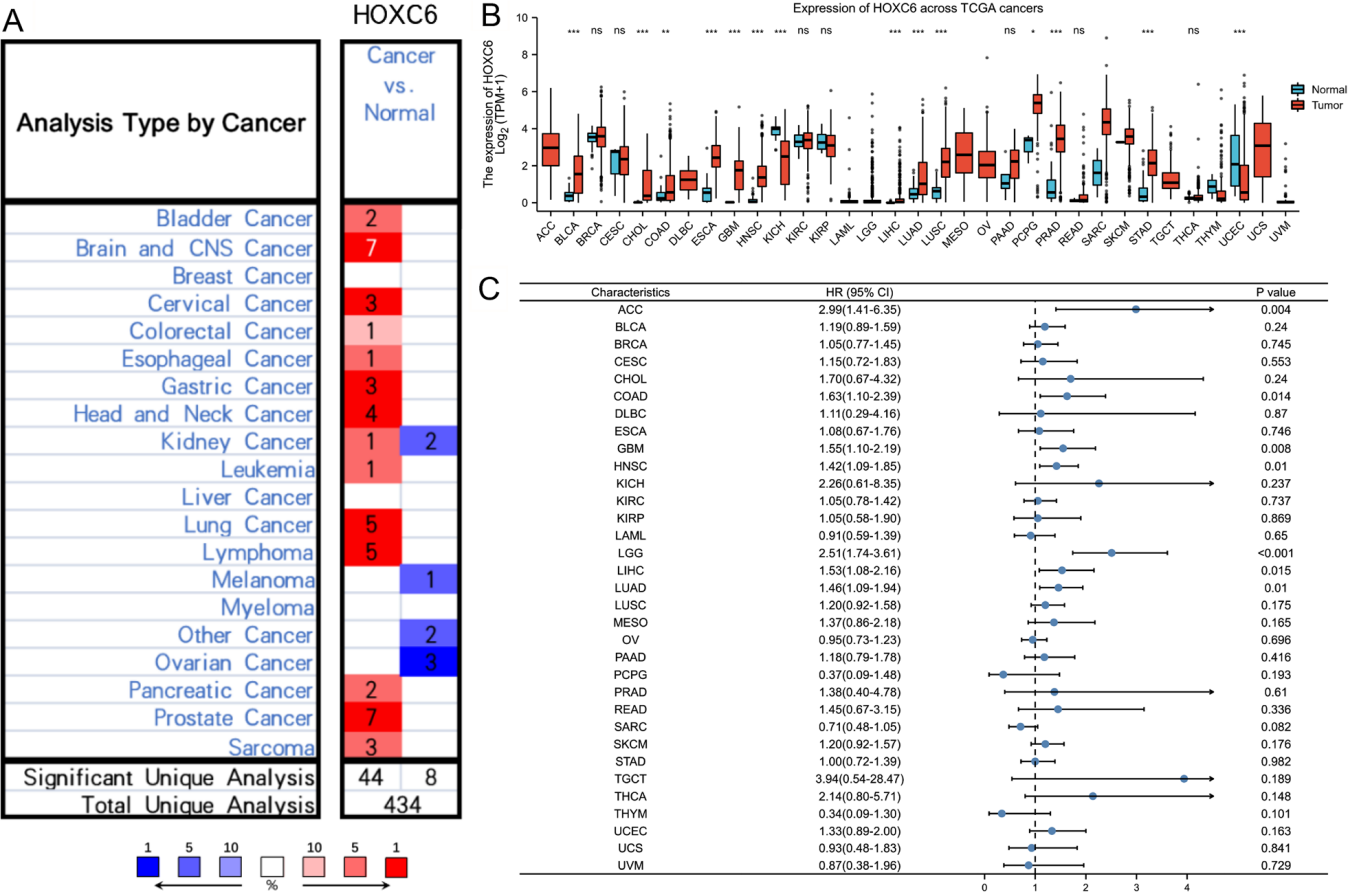
HOXC6 expression was upregulated in multiple cancers. **A** Oncomine analysis of HOXC6 expression in human cancers. The search thresholds are as follows: |fold change|>1.5, P value < 0.001, and top 10% gene ratio. **B** HOXC6 expression information in normal tissues and tumour tissues from multiple cancers in TCGA and GTEx. **C** The forest plot showed that HOXC6 was an independent risk factor for patients with some cancers

Differences in HOXC6 expression between tumour and normal tissues from patients with different cancers were detected by using data from the Oncomine database. HOXC6 mRNA was overexpressed in many cancers, especially in brain and CNS cancer, lung cancer, lymphoma and prostate cancer. In addition, the expression of HOXC6 in ovarian cancer and melanoma was low (Fig. 1A). According to the TCGA and GTEx databases, HOXC6 expression was further analysed and found to be upregulated in most types of tumours but reduced in Kidney Chromophobe (KICH) and Uterine Corpus Endometrial Carcinoma (UCEC) (Fig. 1B). As shown in Fig. 1C, HOXC6 was considered a risk factor for Glioblastoma multiforme (GBM), Brain Lower Grade Glioma (LGG), Adrenal Cortical Carcinoma (ACC), Colon Adenocarcinoma (COAD), Head and Neck Squamous Cell Carcinoma (HNSC), Liver Hepatocellular Carcinoma (LIHC) and Lung Adenocarcinoma (LUAD) with p values of less than 0.05. Furthermore, we analysed the significant changes in HOXC6 expression at the transcriptional level between different forms of brain and CNS cancers vs. normal brain tissues using the Oncomine database (Table 1). Generally, HOXC6 plays a significant role in brain and CNS cancers. The abbreviations for each cancer are summarized in Additional file 2: Table S1.

**Fig. 2:**
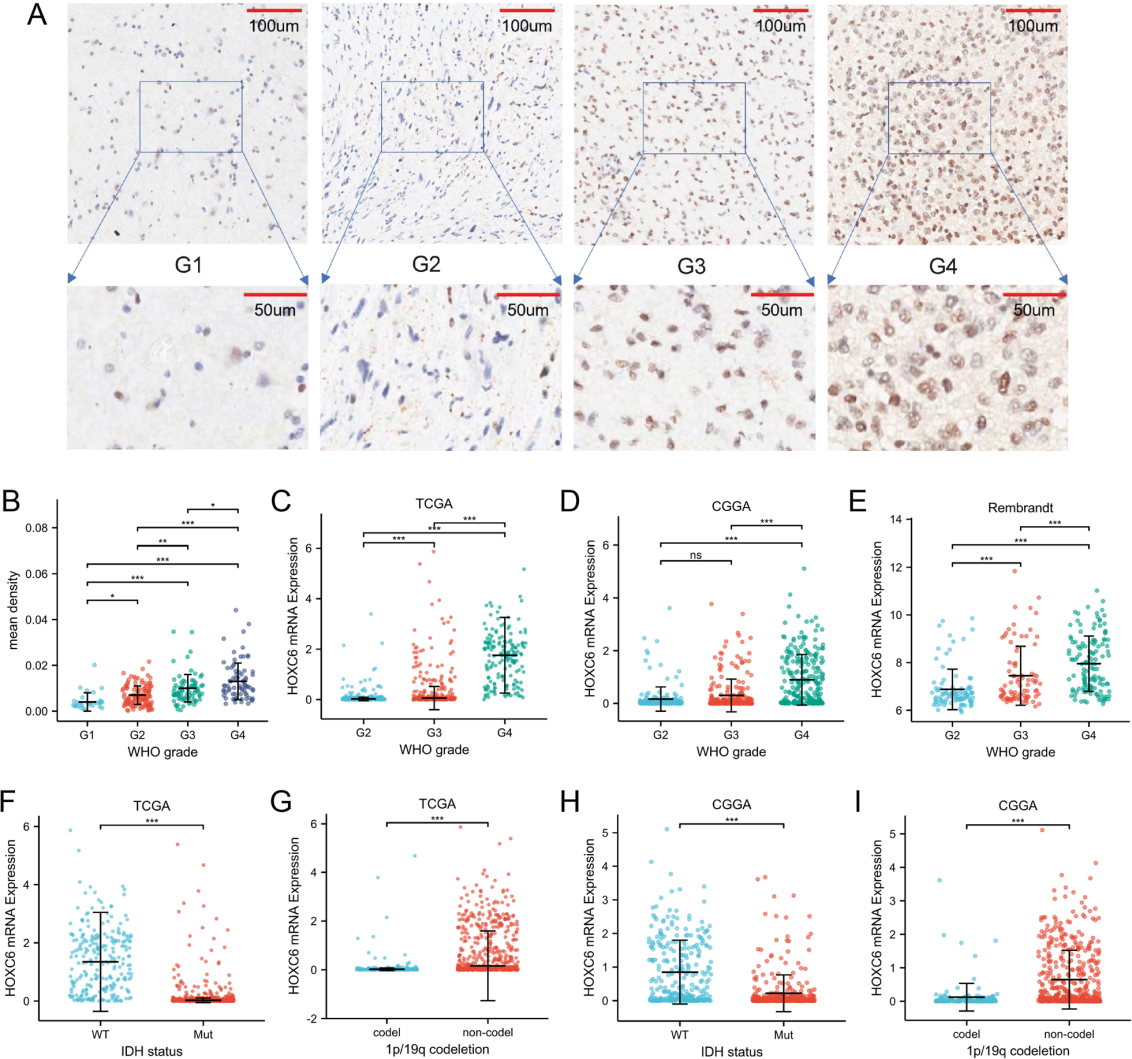
HOXC6 was overexpressed in gliomas and correlated with clinical features. **A** Representative IHC staining of HOXC6 in WHO grade I-IV gliomas. **B** Mean density of HOXC6 in gliomas with different WHO grades. **C**-**E** High HOXC6 expression was positively correlated with high-grade gliomas in the TCGA, CGGA and Rembrandt datasets. **F**-**G** HOXC6 was significantly overexpressed in wild-type (WT) IDH gliomas based on the TCGA and CGGA datasets. **H**-**I** HOXC6 was upregulated in the 1p/19q non-codeletion group based on the TCGA and CGGA datasets. (* mean p < 0.05, ** mean p < 0.01, *** mean p < 0.001, **** mean p < 0.0001)

Considering the emerging role of epigenetic regulators as targets for cancer therapy, we tested whether HOXC6 was differentially expressed in the context of gliomas. For this, we analysed the protein expression level of HOXC6 in glioma tissues in 299 patient samples using IHC, which verified that the protein expression of HOXC6 was positively correlated with a high grade of glioma (Fig. 2A, B). HOXC6 expression correlated positively with glioma grade in the TCGA dataset (Fig. 2C). Consistent with the TCGA database, the HOXC6 expression data in the CGGA and Rembrandt databases showed the same tendency (Fig. 2D and E). Here, we subdivided the TCGA samples into gliomas with a wild-type isocitrate dehydrogenase (IDH) gene, with a mutant-type IDH gene, with 1p/19q codeletion, and without 1p/19q codeletion. According to the TCGA database, gliomas with a wild-type IDH gene and gliomas without 1p/19q codeletion had a high level of HOXC6 expression consistent with the group of gliomas with a mutant-type IDH gene and with 1p/19q codeletion (Fig. 2F, H). Consistent with the TCGA database, the expression of HOXC6 in CGGA showed a consistent tendency (Fig. 2G and I).

### Correlation between HOXC6 expression and prognosis in patients with gliomas

**Fig. 3:**
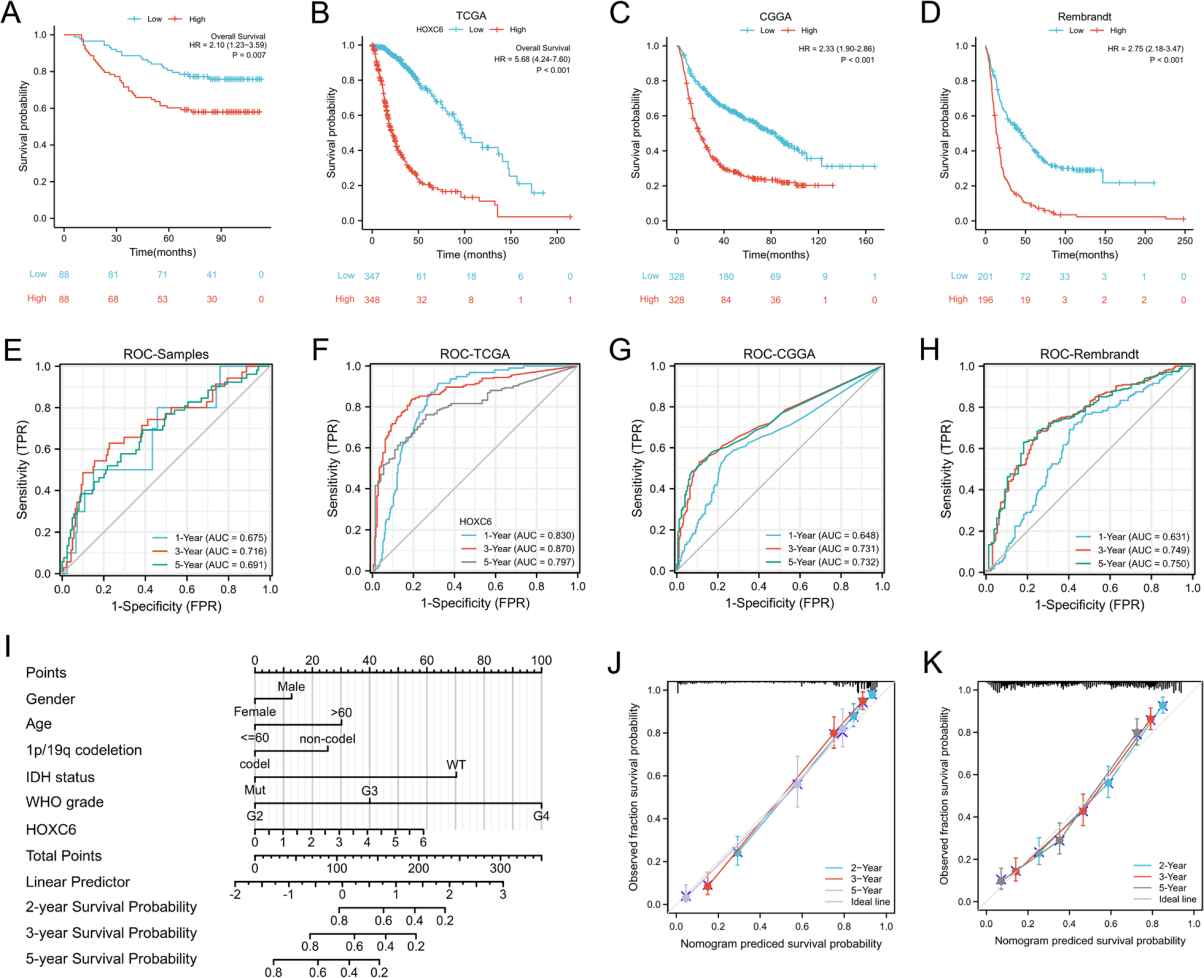
HOXC6 predicts a poor prognosis in glioma patients. **A**–**D** Higher HOXC6 expression portended shorter OS in patients with gliomas based on clinical samples (n = 176) and the TCGA (n = 695), CGGA (n = 656) and Rembrandt datasets (n = 397). **E**–**H** ROC curves based on the above samples. I, Nomogram for predicting 2-, 3- and 5-year survival in glioma patients based on the TCGA dataset. **J**–**K** Calibration curves were used to predict the 2-, 3-, and 5-year survival in the TCGA and CGGA datasets

The relationship between HOXC6 expression and prognosis in patients with gliomas was further analysed using our clinical samples, which illustrated that the high expression of HOXC6 was related to a short OS (Fig. 3A). Additionally, we found that low HOXC6 expression was associated with a longer OS in the TCGA database (Fig. 3B), which was consistent with the CGGA and Rembrandt databases (Fig. 3C, D).

Then, we structured the ROC curve on account of the clinical samples, TCGA, CGGA and Rembrandt datasets to explore the value of HOXC6 for glioma diagnosis according to the area under the ROC curve (AUC). All the above ROC curves verified the high diagnostic value of HOXC6 for the survival prognosis of glioma (Fig. 3E–H).

The prognostic nomogram with a risk classification system for 2-, 3- and 5-year survival rates of glioma based on TCGA was performed (n = 703, Fig. 3I). To test the efficiency of the new nomogram, 200 bootstrap re-samplings were performed for internal verification through the calibration chart in the two independent cohorts of TCGA and CGGA, which indicated a good calibration effect of the nomogram (Fig. 3J, K). Univariate (HR = 5.678, p < 0.001) and multivariate (HR = 1.649, p = 0.022) Cox regression analyses were then conducted, and factors related to the prognosis of glioma were chosen (Table 2). Baseline clinical data in the TCGA, CGGA datasets and clinical patients can be found in Additional file 5: Table S4, Additional file 6: Table S5, Additional file 7: Table S6.

**Table 2:** Univariate and multivariate analyses of overall survival based on TCGA data

Characteristic	Total (N)	Univariate analysis		Multivariate analysis	
		Hazard ratio (95% CI)	P value	Hazard ratio (95% CI)	P value
Sex	695				
Female	297	Reference			
Male	398	1.262 (0.988–1.610)	0.062	1.253 (0.951–1.652)	0.109
Age	695				
<=60	552	Reference			
> 60	143	4.668 (3.598–6.056)	**< 0.001**	1.502 (1.104–2.043)	**0.010**
1p/19q codeletion	688				
Codel	170	Reference			
Non-codel	518	4.428 (2.885–6.799)	**< 0.001**	1.342 (0.806–2.234)	0.258
IDH status	685				
WT	246	Reference			
Mut	439	0.117 (0.090–0.152)	**< 0.001**	0.350 (0.228–0.536)	**< 0.001**
WHO grade	634				
G2	223	Reference			
G3	243	2.999 (2.007–4.480)	**< 0.001**	1.824 (1.184–2.810)	**0.006**
G4	168	18.615 (12.460-27.812)	**< 0.001**	4.200 (2.474–7.130)	**< 0.001**
HOXC6	695				
Low	347	Reference			
High	348	5.678 (4.243–7.598)	**< 0.001**	1.649 (1.076–2.529)	**0.022**

**Fig. 4:**
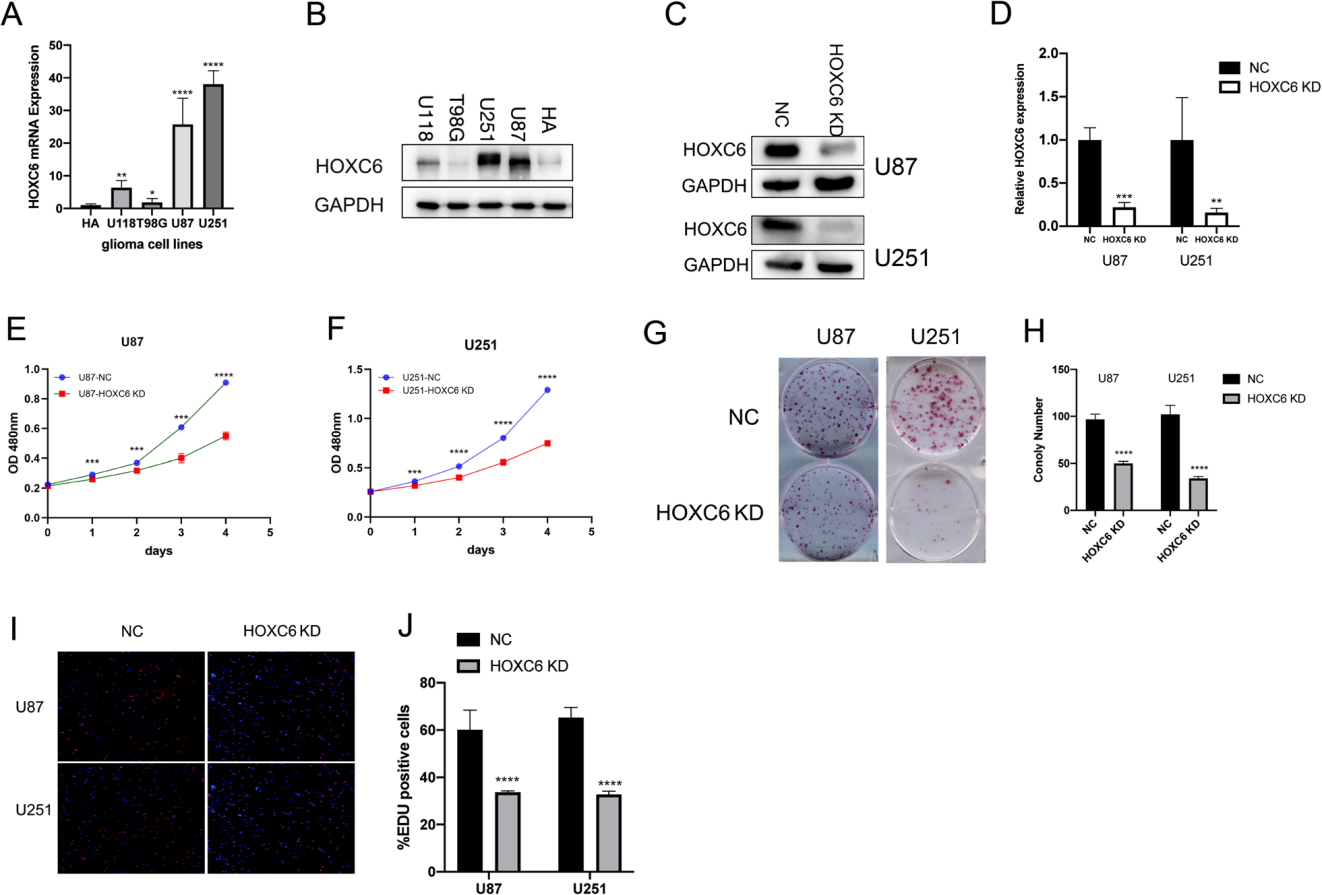
HOXC6 promotes proliferation of glioma cells. **A**, **B** Western blot and RT q-PCR analysis of HOXC6 expression in various types of glioma cells as indicated. GAPDH was used as an internal control. **C**, **D** Verification of HOXC6 knockdown in U87 and U251 cells by Western blotting and q-PCR. **E**, **F** A CCK-8 assay was performed in U87 and U251 cells transfected with control shRNA and HOXC6 shRNA (n = 3, p < 0.05). **G**, **H** Colony-formation assays conducted in U87 and U251 cells transfected with control shRNA and HOXC6 shRNA (n = 3, p < 0.05). **I**, **J** EdU assays were performed in U87 and U251 cells transfected with control shRNA and HOXC6 shRNA (n = 3, p < 0.05). (* mean p < 0.05, ** mean p < 0.01, *** mean p < 0.001, **** mean p < 0.0001)

Subsequently, we conducted verification tests to verify that HOXC6 upregulation is strongly associated with poor prognosis in patients with gliomas. Western blot and RT qPCR showed varied expression levels of GATA3 in U118, T98G, U251, U87 and HA cells (Fig. 4A, B). After transfection in U251 and U87 cells, HOXC6 expression was drastically decreased (Fig. 4C, D). Cell proliferation assays, including the CCK-8 assay (Fig. 4E, F), colony-forming assay (Fig. 4G, H) and EdU assay (Fig. 4I, J), were performed using the transfected cells. All these assays revealed that HOXC6 notably promotedcell proliferation in glioma cells, which demonstrated the correlation between low HOXC6 expression and good prognosis in gliomas.

### HOXC6-related EMT-associated tumour invasion and metastasis in gliomasHOXC6-related EMT-associated tumour invasion and metastasis in gliomas

**Fig. 5:**
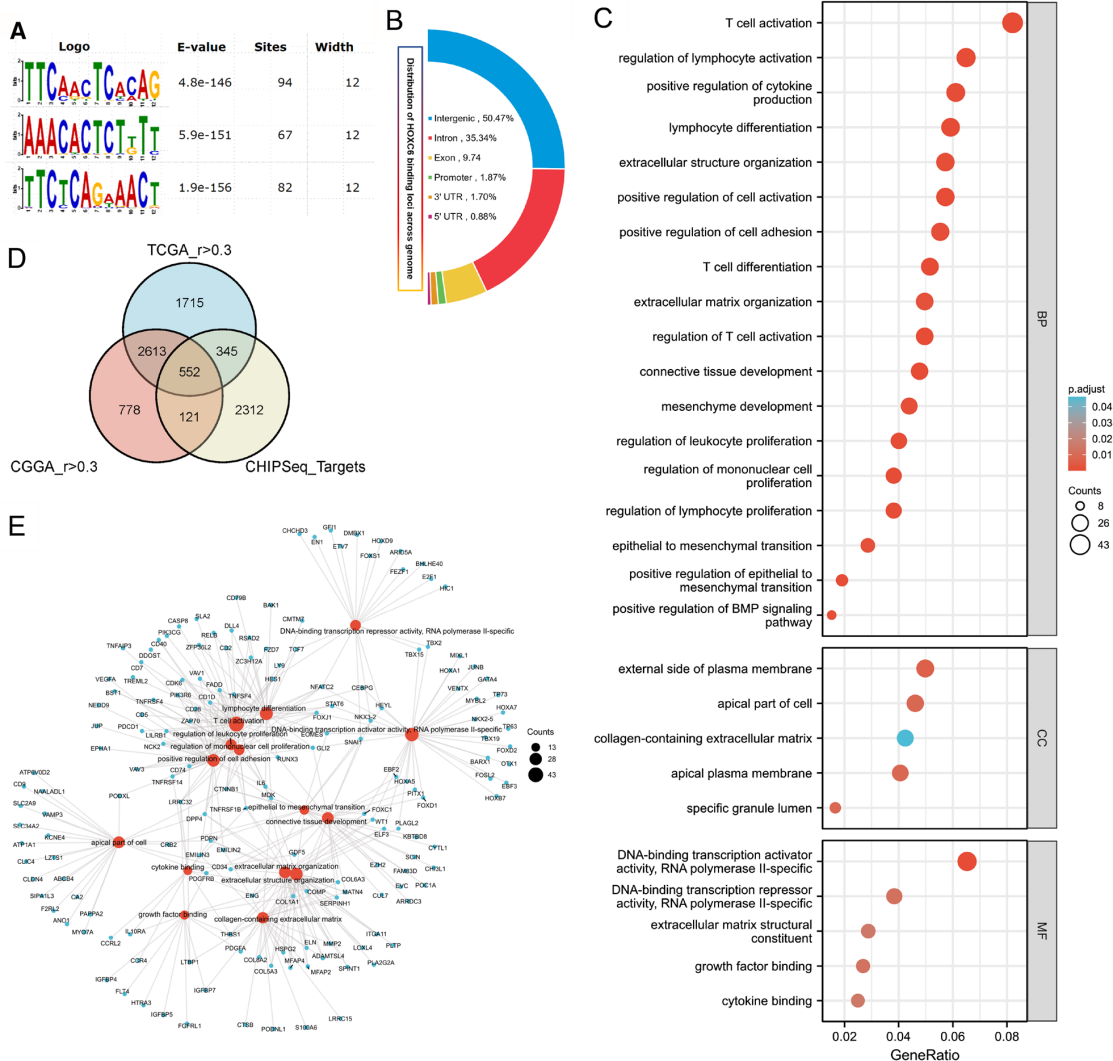
HOXC6-related biological processes in gliomas. **A** Conserved motifs identified by HOXC6 ChIP-seq. **B** Distribution of HOXC6 loci across the genome. **C** Representative GO analysis results. **D** Venn diagram for HOXC6 target genes from ChIP-seq and positive HOXC6-related genes in the TCGA and CGGA databases (Spearman’s R > 0.3, p < 0.05). **E** Network diagram of enrichment map results

According to the aforementioned results, HOXC6 may have an important impact on the biological functions of gliomas. Since HOXC6 is a significant transcription factor in many tumours, ChIP-seq was performed. The HOXC6-bound DNA motifs were identified by using MEME software (Fig. 5A), and the distribution of HOXC6 peak binding locations across the genome is shown in Fig. 5B. To further clarify the biological roles of HOXC6 expression in glioma, we selected 5225 genes from the TCGA dataset (Spearman’s R > 0.3, p < 0.05), 4064 genes from the CGGA dataset (Spearman’s R > 0.3, p < 0.05) and 3330 target genes from ChIP-seq (Fig. 5C). Then, the intersection of genes selected in the three datasets was explored by GO analysis. We found that the most involved terms were EMT, positive regulation of cell adhesion, extracellular matrix organization, positive regulation of EMT, T-cell activation, T-cell differentiation and so on when the gene functions were sorted by p-adjust < 0.05 and FDR < 0.2 (Fig. 5D). The network diagram of the enrichment map showed that the enriched terms were centrally concentrated in EMT-related pathways as well as the immune response (Fig. 5E).

**Fig. 6:**
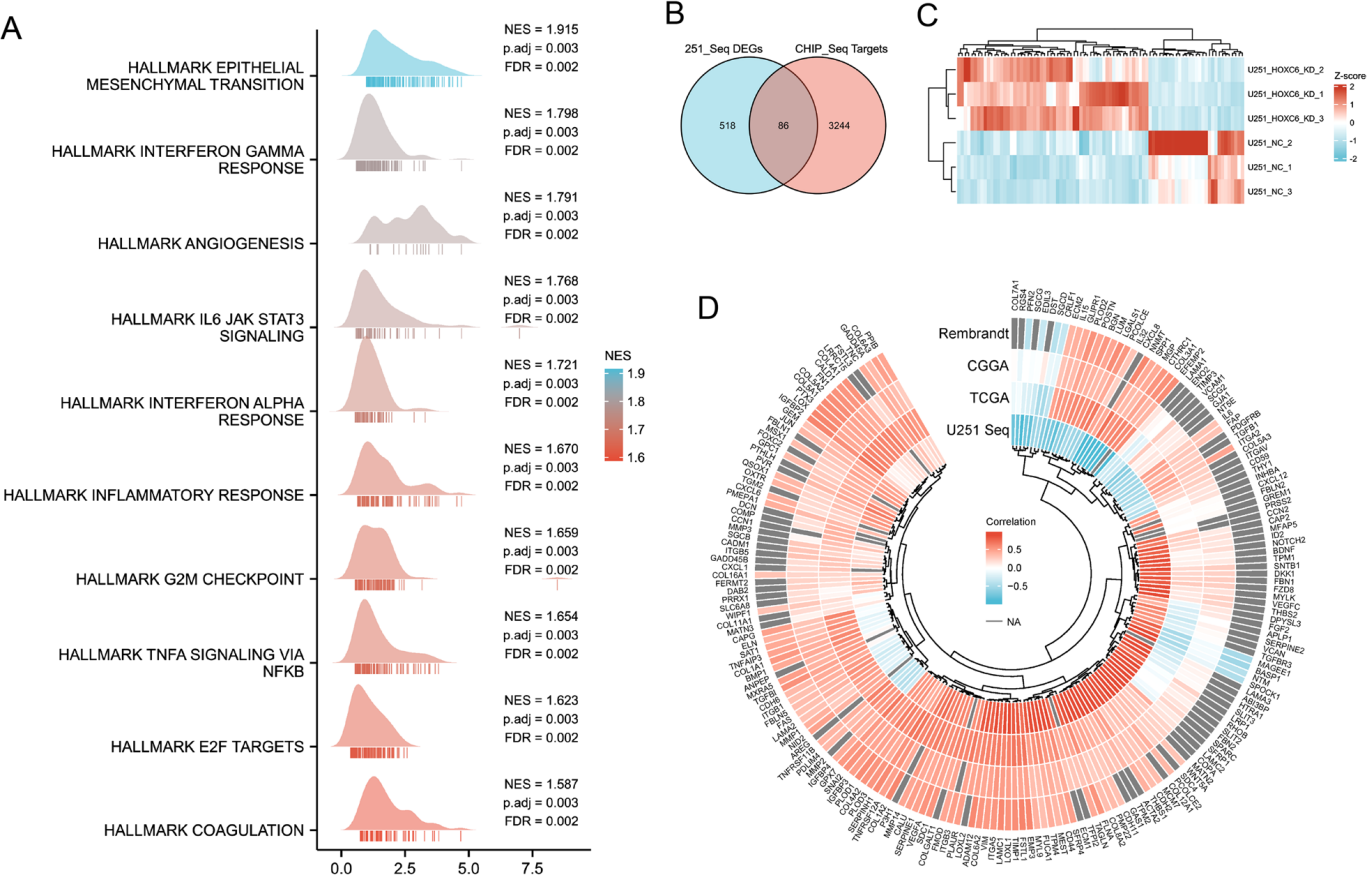
HOXC6 promotes EMT processes. **A** Ridge plot to verify the gene signatures. **B** Venn diagram indicating the intersection of genes in RNA-seq and ChIP-seq. **C** Heatmap cluster based on the ChIPseq annotated targets differentially expressed in the sequencing results at the thresholds | fold change | > 2 and p value < 0.05. **D** Circular heatmap showing Spearman’s correlation between HOXC6 expression and the expression of EMT-related biomarkers based on the RNA-seq, TCGA, CGGA and Rembrandt databases

After GSEA, it was easy to find that EMT-related pathways and inflammatory responses were positively affected by HOXC6 using the ridge plot (Fig. 6A). Then, we used a Venn diagram to indicate the intersection of HOXC6 differentially regulated genes and ChIP-seq annotated targets (Fig. 6B). We listed the ChIP-seq annotated targets differentially expressed in the sequencing results at the thresholds | fold change | > 2 and p value < 0.05 (Fig. 6C). Sequencing data on EMT-related genes and ChIP-seq annotated targets can be found in Additional file 8: Table S7. In addition,circular heatmap based on the RNA-seq, TCGA, CGGA and Rembrandt databases demonstrated that HOXC6 expression levels had a positive relationship with most EMT-related biomarkers (Fig. 6D).

### HOXC6 is involved in the regulation of the Tumour Immune MicroenvironmentHOXC6 is involved in the regulation of the Tumour Immune Microenvironment

**Fig. 7:**
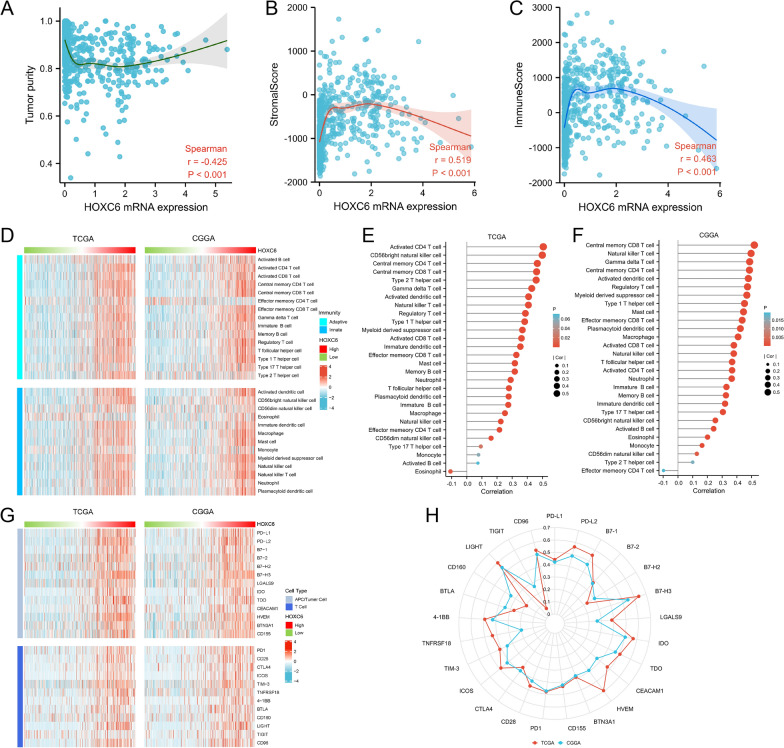
HOXC6 is involved in regulation of the tumour immune microenvironment. **A** HOXC6 expression is negatively related to tumour purity. **B** The expression of HOXC6 is positively related to the stromal score. **C** HOXC6 expression is positively related to the immune score. **D**-**E** The heatmap and the lollipop chart based on the TCGA and CGGA databases illustrate the relationship between HOXC6 and 28 infiltrating immune cell populations. **F**-**G** The heatmap and the radar chart based on the TCGA and CGGA databases illustrate the relationship between HOXC6 and immune checkpoint markers

Since previous experimental data showed a relationship between HOXC6 expression and glioma biological progression as well as the immune response, we further considered whether the expression of HOXC6 was relevant to the formation of the tumour immune microenvironment. To test this hypothesis, we first analysed the relationship between the HOXC6 expression level and tumour purity, which showed a negative association (Fig. 7A). Then, the scatter diagram was displayed to show the positive association between HOXC6 expression and the stromal score (Fig. 7B). In addition, we investigated the relationship between HOXC6 expression and the immune score, which remained positive (Fig. 7C). The above results demonstrate that HOXC6 expression has a close relationship with the tumour immune microenvironment.

Hence, we next used immune cell GSVA to assess whether certain immune infiltrates might be associated with elevated HOXC6 expression in TCGA and CGGA. A heatmap was used to show the relationship between the expression level of HOXC6 and 28 infiltrating immune cell features based on the TCGA and CGGA databases (Fig. 7D). Most immune infiltration levels in the HOXC6 high-expression group were higher than those in the HOXC6 low-expression group. The lollipop diagram showed the ordering of HOXC6 in relation to these immune cell infiltrates (Fig. 7E). Detailed correlations of HOXC6 expression levels with the mentioned infiltrating immune cell features are shown in Additional file 3: Table S2.

The association between HOXC6 and some important immune checkpoint proteins, including PD-L1, PD-1, IDO, TDO, B7-H3, CTLA-4, and LGALS9, was further explored. Heatmaps showed that upregulation of HOXC6 was positively correlated with these immune checkpoint proteins according to the TCGA and CGGA databases (Fig. 7F). The radar map detailed the positive correlation between HOXC6 and most common checkpoint proteins. Among these immune checkpoint proteins, B7-H3 had the strongest positive association with HOXC6 (Fig. 7G). In general, most of these important checkpoint proteins indicated a significant positive association with HOXC6 expression. Detailed correlations of HOXC6 expression levels with the mentioned immune checkpoints are shown in Additional file 4: Table S3.

**Fig. 8:**
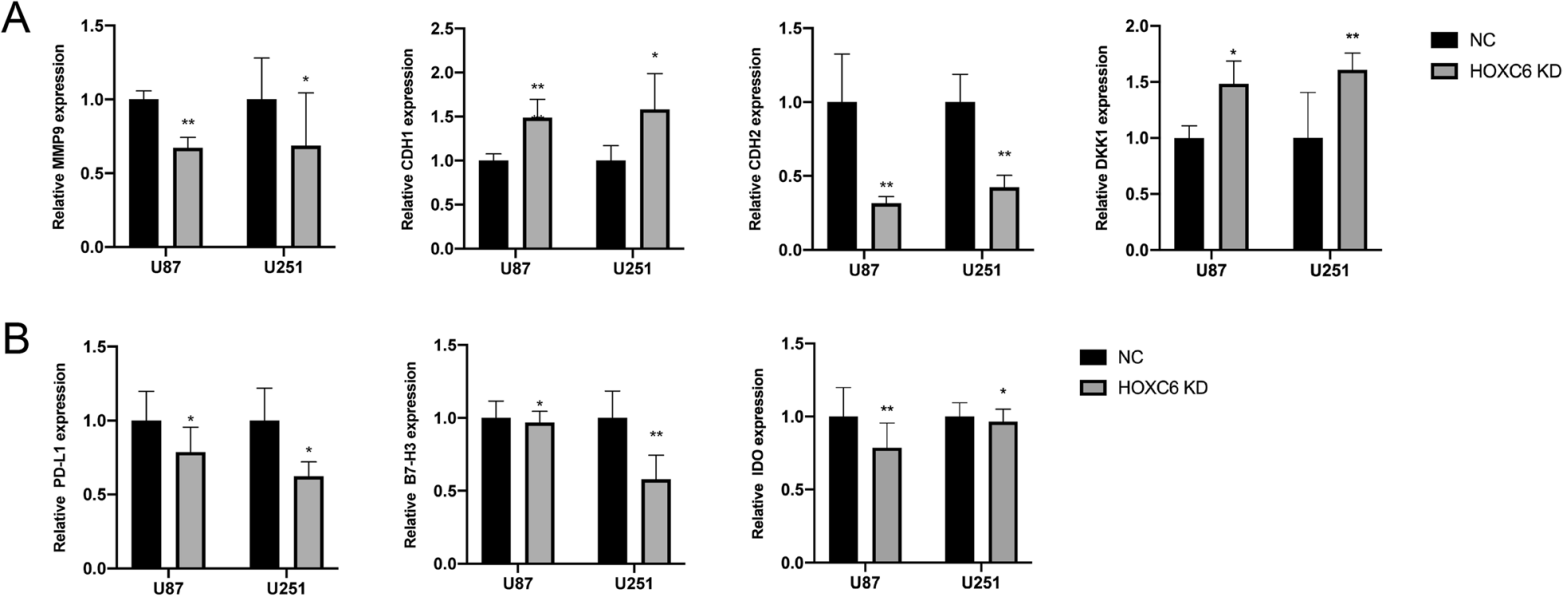
Verification of the positive correlation between HOXC6 expression and the mentioned genes. **A** The RT q-PCR analysis of HOXC6-correlated EMT-associated genes. **B** RT q-PCR analysis of HOXC6-correlated immune checkpoint proteins

To identify the positive relationship between HOXC6 expression and EMT-related genes, we performed RT q-PCR assays (Fig. 8A). The results confirmed the positive association between HOXC6 expression and the EMT-related genes we analysed above. Then, we conducted RT q-PCR assays to verify the positive correlation between the expression of HOXC6 and the abovementioned immune checkpoint proteins (Fig. 8B). As we expected, a positive significant association between HOXC6 and key immune checkpoint proteins was shown.

## Discussion

Despite great progress in glioma surgery, radiotherapy [22] and chemotherapy [23], the prognosis of glioma patients is still poor. Genetic examination can be used to guide radiotherapy and chemotherapy for glioma. For instance, patients with mutations in IDH1 and IDH2 have a better prognosis and clinical response after radiotherapy and chemotherapy [24, 25]. In addition, people with 1p19q non-codeletion were considered insensitive to radiotherapy [26, 27]. Consequently, there is an urgent need for more therapeutic targets and molecular markers.

HOXC6 is a member of the HOXC cluster, located on chromosome 12Q13.13. The protein encoded by HOXC6 consists of 235 amino acids, of which amino acids 145–198 constitute the classical homeodomain domain that mediates HOXC6 binding to the target gene promoter region. However, the function of regions 1-144 and 199–235 is unclear [28]. Therefore, we used ChIP-Seq to detect its target genes to analyse its biological function. Herein, we demonstrated that HOXC6 was overexpressed in many cancers, especially in glioma. Moreover, we found that HOXC6 was significantly upregulated in high-grade glioma, a wild-type IDH gene and 1p19q non-codeletion, and these characteristics indicate tumour malignancy and are insensitive to chemotherapy [29].

In a previous study, high HOXC6 expression resulted in poor survival in patients with multiple cancers [7, 30]. As expected, our study showed that patients with high levels of HOXC6 tended to have a shorter OS than those with low levels in clinical samples and the TCGA, CGGA and Rembrandt datasets. By assessing individualized prognostic forecasts in the nomogram [31], we confirmed HOXC6 as a prognostic biomarker of glioma. The calibration plots of the two datasets were highly fitted, illustrating that the nomogram performed well in predicting 2-, 3- and 5-year survival in patients with gliomas. Therefore, we inferred that HOXC6 can act as a predictor for the clinical prognosis of glioma patients.

To further explore the biological function of HOXC6 in glioma, GO analyses were conducted to reveal that HOXC6 was strongly associated with the functions of tumour progression, apoptosis, EMT and inflammation. GSEA further revealed significant enrichment of EMT, angiogenesis, inflammatory response, and the IFN-α pathway, IFN-γ pathway and IL6/JAK/STAT3 pathway. The functional network binding to hub genes indicated that the EMT signalling pathway may be the core pathway by which HOXC6 regulates tumour progression.

EMT is an important process in which epithelial cells lose cell polarity and adhesion and become mesenchymal cells. This is closely related to the existence of glioma stem cells discovered in stem cell research in recent years and ultimately leads to a high capacity for migration and invasion, resistance to radiotherapy and chemotherapy and tumour recurrence [32]. As reported in previous studies of other types of cancer, HOXC6 exhibited the biological function of matrix remodelling, cell migration, invasion and metastasis in other types of malignancies, such as laryngeal cancer [33], cervical carcinoma [34] and lung cancer [35]. To further validate the role of HOXC6 in the glioma EMT process, a series of representative genes of the EMT process were selected and analysed to show their high correlation with HOXC6. In previous studies, the interaction of HOXC6 and EMT biomarkers, including MMP9 [36], vimentin [37] and TWIST1 [38], has also been confirmed. In our study, we verified that the expression of MMP9 and CDH2 were decreased, while the expression of CDH1 and DKK1 were increased in HOXC6 knockdown cells. In addition, RNA sequencing further reinforced that knocking down HOXC6 *in vitro* resulted in low expression of EMT-related genes.

In addition, a potential association of EMT with tumour immune environment formation has been shown in recent studies. On the one hand, the cytokines generated upon EMT increase tumour infiltration by immune cells [39]. On the other hand, the EMT process may weaken the actions of immune effector cells via regulator T cells (Tregs) and interferon gamma signals, leading to an escape from recognition and killing by the immune system [40]. These findings may partly account for the results of our study: HOXC6 expression is positively correlated with the stromal and immune scores of glioma patients but negatively correlated with tumour purity. Thus, our findings might illustrate the potential dual value of HOXC6 both in EMT targeting and in immunological therapy.

Moreover, gene set variation analysis indicated that there was a close link between HOXC6 and infiltrating immune cells, including immunosuppressive components such as Tregs and myeloid-derived suppressor cells (MDSCs). Several studies have demonstrated that the proportion of Tregs increases with the degree of glioma malignancy [41]. Tregs suppress T-cell proliferation by capturing IL2 and regulate the function of effector T cells by secreting inhibitory cytokines, including IL-10 [42]. More importantly, CTLA-4 on Treg cells downregulates the expression of CD80 and CD86 on DCs, thereby inducing DC tolerance, which further inhibits the ability of T cells to express IDO [43]. In this case, cytokines produced by MDSCs and Tregs form a positive feedback loop to enhance immunosuppression. The TCGA and CGGA data analysis showed that HOXC6 is strongly linked to CTLA-4, IDO, and TDO. Therefore, HOXC6 is very likely to promote the functions of these tumour-infiltrating immunosuppressive cells.

In recent decades, checkpoint inhibitors have achieved remarkable results in tumour treatment and have become the most advanced immunotherapy in clinical application [44]. As the most-studied checkpoint, PD-L1 involves a complex mechanism, and its important relationship with interferon has been revealed in previous studies. PD-L1 is widely expressed in glioma, participates in tumour-induced immunoregulation of infiltrating T cells and plays the role of a negative prognostic factor [45]. As important cytokines secreted by T cells, interferons have a dual role in the tumour process. Interferons can activate DCs to release tumour necrosis factor-related apoptosis-inducing ligands, thereby strengthening the functions of tumour-infiltrating natural killing cells and cytotoxic T lymphocytes (CTLs) [46]. Nevertheless, in some cases, interferons upregulate the expression of PD-L1 on the tumour cell surface and enhance the infiltration of immunosuppressive cells, leading to the inhibition of the antitumor immune response and the occurrence of tumour immune escape [47]. In our study, the Spearman correlation coefficients between HOXC6 and PD-L1 were 0.44 in TCGA gliomas and 0.42 in CGGA gliomas. GSEA showed a clear enrichment of interferon signalling pathways. These results indicate that HOXC6 expression in glioma patients may predict the therapeutic efficacy of PD-L1 blocker therapy.

In addition, using the TCGA and CGGA datasets, we demonstrated that B7-H3 showed the strongest correlation with HOXC6 among the members of the B7 ligand family. B7-H3 is associated with poor prognosis and is a valuable guide for immunotherapy. In malignant tissues, B7-H3 inhibits tumour antigen-specific immune responses, leading to tumorigenic effects [48]. B7-H3 also has non-immuno-pro-tumour effects, such as in angiogenesis, drug resistance, EMTEMT and influencing tumour cell metabolism [49]. In our study, the Spearman correlation coefficients between HOXC6 and B7-H3 were 0.64 in TCGA gliomas and 0.55 in CGGA gliomas. HOXC6 exhibited widespread correlations with other immune checkpoints, such as LGALS9 and CD96. PD-L1 and B7-H3 expression levels were decreased in HOXC6 knockdown glioma cell lines. These results illustrate the extremely broad and promising applications of HOXC6 for immune checkpoint treatments.

Of course, there are some limitations in our research. First, although our results are based on a large number of bioinformatics analyses, RNA-seq and ChIP-seq, the specific in vivo mechanisms of how HOXC6 regulates immunity and EMT still need to be further explored. Second, we also need more animal models to validate our findings and advance the clinical application of HOXC6 as a glioma immunotherapy target.

## Conclusions

In conclusion, HOXC6 is a prognostic indicator in glioma. Moreover, our study broadens our understanding of the functions of HOXC6 transcription target genes. HOXC6, which is associated with EMT and the immune microenvironment, is expected to be a potential therapeutic target for glioma. These findings may lead to new perspectives on the treatment of gliomas. We provide a flow chart to  better reflect the design of our bioinformatics investigation in Additional file 1: Figure S1.

## Supplementary Information

**Additional file 1:** **Figure S1** A flow chart to  better reflect the design of our bioinformatics investigation.

**Additional file 2:** **Table S1** The abbreviations for mentioned cancers.

**Additional file 3:** **Table S2** The detailed correlations of HOXC6 expression levels with the mentioned infiltrating immune cell features.

**Additional file 4:** **Table S3** The detailed correlations of HOXC6 expression levels with the mentioned immune checkpoints.

**Additional file 5:** **Table S4** Baseline clinical data of TCGA gliomas.

**Additional file 6:** **Table S5** Baseline clinical data of CGGA gliomas.

**Additional file 7:** **Table S6** Baseline clinical data of patient samples.

**Additional file 8:** **Table S7** The sequencing data on EMT-related genes and ChIP-seq annotated targets.

## Data Availability

The datasets in our study are available in the UCSC XENA public repository (https://xenabrowser.net/datapages/), CGGA database (http://www.cgga.org.cn/) and Rembrandt database supplied by gliovis (http://gliovis.bioinfo.cnio.es/).
