# Supplementary figures and images for: HOXC6 impacts epithelial-mesenchymal transition and the immune microenvironment through gene transcription in gliomas
Source: Cancer Cell Int. 2022 Apr 29;22:170. doi: 10.1186/s12935-022-02589-9 (PMC9052479; doi:10.1186/s12935-022-02589-9)

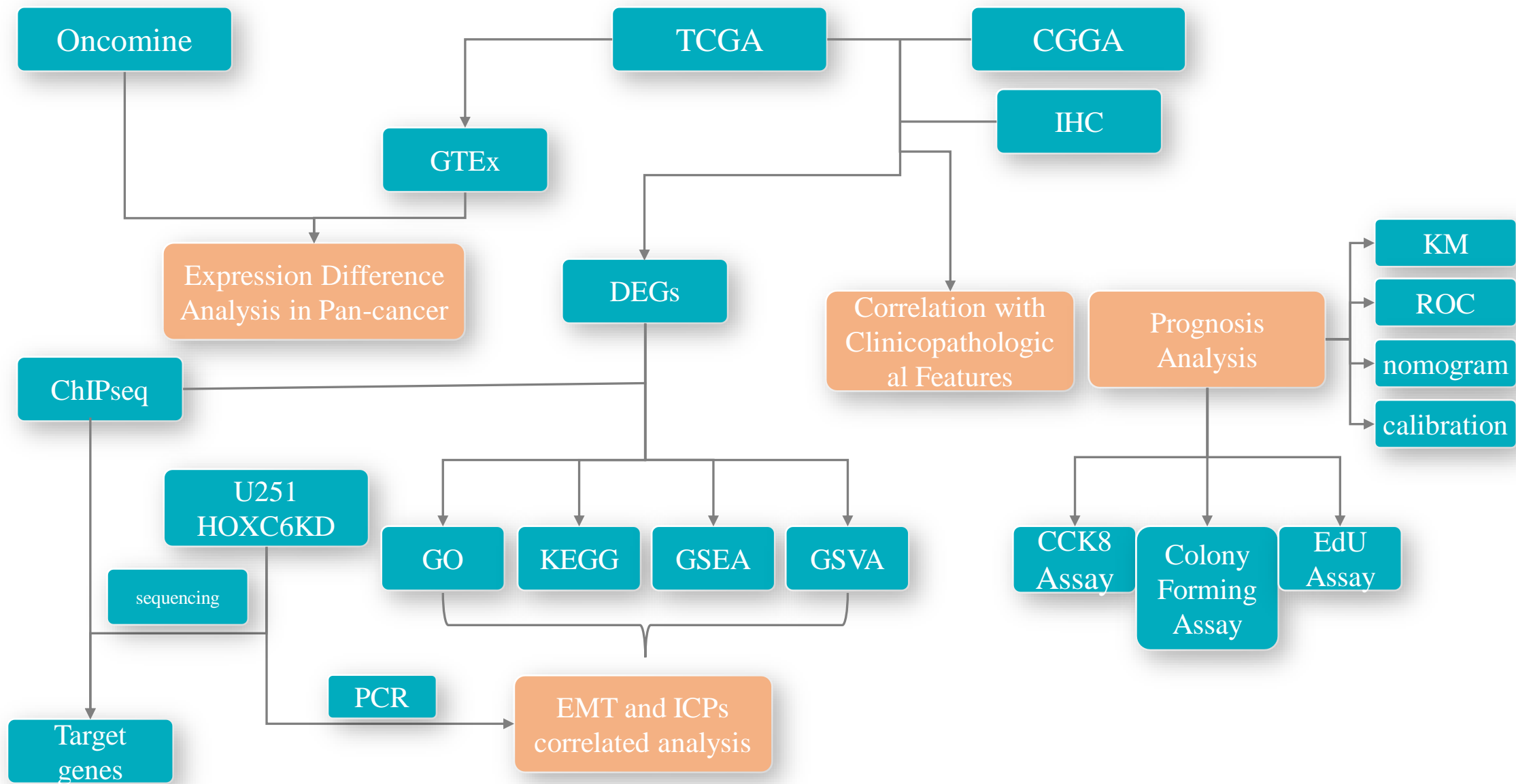

Supplement: Supplementary file 1 — Additional file 1: Figure S1 A flow chart to better reflect the design of our bioinformatics investigation. [file 12935_2022_2589_MOESM1_ESM.pdf]
