# Supplementary material for: HOXC6 impacts epithelial-mesenchymal transition and the immune microenvironment through gene transcription in gliomas
Source: Cancer Cell Int. 2022 Apr 29;22:170. doi: 10.1186/s12935-022-02589-9 (PMC9052479; doi:10.1186/s12935-022-02589-9)
Supplement: Supplementary file 3 — Additional file 3: Table S2 The detailed correlations of HOXC6 expression levels with the mentioned infiltrating immune cell features. [file 12935_2022_2589_MOESM3_ESM.docx]

|  | TCGA | | CGGA | |
| --- | --- | --- | --- | --- |
| HOXC6 | correlation | pvalue | Correlation | pvalue |
| Activated B cell | 0.461702481 | *** | 0.518391858 | *** |
| Activated CD4 T cell | 0.407346767 | *** | 0.496311595 | *** |
| Activated CD8 T cell | 0.429479697 | *** | 0.486828864 | *** |
| Activated dendritic cell | 0.466326706 | *** | 0.485474333 | *** |
| CD56bright natural killer cell | 0.408390999 | *** | 0.477944735 | *** |
| CD56dim natural killer cell | 0.387517428 | *** | 0.472306654 | *** |
| Central memory CD4 T cell | 0.367728244 | *** | 0.467646023 | *** |
| Central memory CD8 T cell | 0.382826644 | *** | 0.451779022 | *** |
| Effector memeory CD4 T cell | 0.317764711 | *** | 0.444983551 | *** |
| Effector memeory CD8 T cell | 0.327496476 | *** | 0.435978433 | *** |
| Eosinophil | 0.276586935 | *** | 0.423774668 | *** |
| Gamma delta T cell | 0.252286088 | *** | 0.408294375 | *** |
| Immature B cell | 0.361596689 | *** | 0.379465959 | *** |
| Immature dendritic cell | 0.224645887 | *** | 0.37872599 | *** |
| Macrophage | 0.278527368 | *** | 0.367551928 | *** |
| Mast cell | 0.505720145 | *** | 0.367406347 | *** |
| Memory B cell | 0.289811903 | *** | 0.36498518 | *** |
| Monocyte | 0.27396945 | *** | 0.329182682 | *** |
| Myeloid derived suppressor cell | 0.317180243 | *** | 0.325721283 | *** |
| Natural killer cell | 0.354140396 | *** | 0.319907038 | *** |
| Natural killer T cell | 0.092567874 | * | 0.305201269 | *** |
| Neutrophil | 0.49910649 | *** | 0.253962377 | *** |
| Plasmacytoid dendritic cell | 0.073334303 | 0.070768 | 0.242339683 | *** |
| Regulatory T cell | -0.108186075 | ** | 0.20099749 | *** |
| T follicular helper cell | 0.077250822 | 0.056943 | 0.163598803 | *** |
| Type 1 T helper cell | 0.160455028 | *** | 0.128165242 | ** |
| Type 17 T helper cell | 0.458447094 | *** | 0.100055274 | * |
| Type 2 T helper cell | 0.215796746 | *** | -0.09777941 | * |

(* means p＜0.05, ** means p＜0.01, *** means p＜0.001)
