# Supplementary material for: HOXC6 impacts epithelial-mesenchymal transition and the immune microenvironment through gene transcription in gliomas
Source: Cancer Cell Int. 2022 Apr 29;22:170. doi: 10.1186/s12935-022-02589-9 (PMC9052479; doi:10.1186/s12935-022-02589-9)
Supplement: Supplementary file 4 — Additional file 4: Table S3 The detailed correlations of HOXC6 expression levels with the mentioned immune checkpoints. [file 12935_2022_2589_MOESM4_ESM.docx]

|  | TCGA | | CGGA | |
| --- | --- | --- | --- | --- |
| ICPs | correlation | pvalue | correlation | pvalue |
| PD-L1 | 0.44113412 | *** | 0.421 | *** |
| PD-L2 | 0.56475814 | *** | 0.488 | *** |
| B7-1 | 0.55411618 | *** | 0.47 | *** |
| B7-2 | 0.37690311 | *** | 0.369 | *** |
| B7-H2 | 0.23268576 | *** | 0.304 | *** |
| B7-H3 | 0.63799076 | *** | 0.546 | *** |
| LGALS9 | 0.38578156 | *** | 0.267 | *** |
| IDO | 0.5691701 | *** | 0.504 | *** |
| TDO | 0.50627661 | *** | 0.465 | *** |
| CEACAM1 | 0.47464852 | *** | 0.366 | *** |
| HVEM | 0.59457924 | *** | 0.392 | *** |
| BTN3A1 | 0.39013313 | *** | 0.378 | *** |
| CD155 | 0.4367994 | *** | 0.425 | *** |
| PD1 | 0.48082057 | *** | 0.474 | *** |
| CD28 | 0.46691078 | *** | 0.424 | *** |
| CTLA4 | 0.36231925 | *** | 0.396 | *** |
| ICOS | 0.48864986 | *** | 0.423 | *** |
| TIM-3 | 0.41431898 | *** | 0.348 | *** |
| TNFRSF18 | 0.43678894 | *** | 0.197 | *** |
| 4-1BB | 0.49067014 | *** | 0.427 | *** |
| BTLA | 0.26990407 | *** | 0.346 | *** |
| CD160 | 0.19382105 | *** | 0.344 | *** |
| LIGHT | 0.59757758 | *** | 0.546 | *** |
| TIGIT | 0.06289235 | ** | 0.266 | *** |
| CD96 | 0.53620623 | *** | 0.501 | *** |

(* means p＜0.05, ** means p＜0.01, *** means p＜0.001)
