# Supplementary material for: HOXC6 impacts epithelial-mesenchymal transition and the immune microenvironment through gene transcription in gliomas
Source: Cancer Cell Int. 2022 Apr 29;22:170. doi: 10.1186/s12935-022-02589-9 (PMC9052479; doi:10.1186/s12935-022-02589-9)
Supplement: Supplementary file 5 — Additional file 5: Table S4 Baseline clinical data of TCGA gliomas. [file 12935_2022_2589_MOESM5_ESM.docx]

Baseline clinical data of TCGA gliomas

| Characteristic | Low expression of HOXC6 | High expression of HOXC6 | p |
| --- | --- | --- | --- |
| n | 348 | 348 |  |
| Gender, n (%) |  |  | 0.939 |
| Female | 150 (21.6%) | 148 (21.3%) |  |
| Male | 198 (28.4%) | 200 (28.7%) |  |
| IDH status, n (%) |  |  | < 0.001 |
| WT | 25 (3.6%) | 221 (32.2%) |  |
| Mut | 320 (46.6%) | 120 (17.5%) |  |
| 1p/19q codeletion, n (%) |  |  | < 0.001 |
| codel | 144 (20.9%) | 27 (3.9%) |  |
| non-codel | 204 (29.6%) | 314 (45.6%) |  |
| WHO grade, n (%) |  |  | < 0.001 |
| G2 | 169 (26.6%) | 55 (8.7%) |  |
| G3 | 136 (21.4%) | 107 (16.9%) |  |
| G4 | 1 (0.2%) | 167 (26.3%) |  |
| Age, meidan (IQR) | 38 (31, 48) | 54 (42.5, 63) | < 0.001 |
