# Supplementary material for: HOXC6 impacts epithelial-mesenchymal transition and the immune microenvironment through gene transcription in gliomas
Source: Cancer Cell Int. 2022 Apr 29;22:170. doi: 10.1186/s12935-022-02589-9 (PMC9052479; doi:10.1186/s12935-022-02589-9)
Supplement: Supplementary file 6 — Additional file 6: Table S5 Baseline clinical data of CGGA gliomas. [file 12935_2022_2589_MOESM6_ESM.docx]

**Baseline clinical data of CGGA gliomas**

| Characteristic | Low expression of HOXC6 | High expression of HOXC6 | p |
| --- | --- | --- | --- |
| n | 347 | 346 |  |
| Gender, n (%) |  |  | 1.000 |
| Female | 148 (21.4%) | 147 (21.2%) |  |
| Male | 199 (28.7%) | 199 (28.7%) |  |
| IDH_mutation_status, n (%) |  |  | < 0.001 |
| Mutant | 224 (34.9%) | 132 (20.6%) |  |
| Wildtype | 89 (13.9%) | 197 (30.7%) |  |
| 1p19q_codeletion_status, n (%) |  |  | < 0.001 |
| Codel | 105 (16.9%) | 40 (6.4%) |  |
| Non-codel | 187 (30%) | 291 (46.7%) |  |
| Grade, n (%) |  |  | < 0.001 |
| WHO II | 125 (18.1%) | 63 (9.1%) |  |
| WHO III | 148 (21.4%) | 107 (15.5%) |  |
| WHO IV | 73 (10.5%) | 176 (25.4%) |  |
| Age, meidan (IQR) | 41 (32, 48) | 45 (37.25, 55) | < 0.001 |
