# Supplementary material for: HOXC6 impacts epithelial-mesenchymal transition and the immune microenvironment through gene transcription in gliomas
Source: Cancer Cell Int. 2022 Apr 29;22:170. doi: 10.1186/s12935-022-02589-9 (PMC9052479; doi:10.1186/s12935-022-02589-9)
Supplement: Supplementary file 7 — Additional file 7: Table S6 Baseline clinical data of patient samples. [file 12935_2022_2589_MOESM7_ESM.docx]

**Baseline clinical data of patient samples.**

| Characteristic | Low expression of HOXC6 | High expression of HOXC6 | p |
| --- | --- | --- | --- |
| n | 88 | 88 |  |
| gender, n (%) |  |  | 0.118 |
| Female | 38 (21.6%) | 27 (15.3%) |  |
| Male | 50 (28.4%) | 61 (34.7%) |  |
| WHO grade, n (%) |  |  | < 0.001 |
| G1 | 19 (10.8%) | 4 (2.3%) |  |
| G2 | 48 (27.3%) | 33 (18.8%) |  |
| G3 | 17 (9.7%) | 33 (18.8%) |  |
| G4 | 4 (2.3%) | 18 (10.2%) |  |
| Recurrence, n (%) |  |  | < 0.001 |
| No | 54 (30.7%) | 28 (15.9%) |  |
| Yes | 34 (19.3%) | 60 (34.1%) |  |
| age, mean ± SD | 38.53 ± 17.39 | 45.31 ± 16.47 | 0.009 |
